# Histone Variant H3.3 Mutations in Defining the Chromatin Function in Mammals

**DOI:** 10.3390/cells9122716

**Published:** 2020-12-18

**Authors:** Matteo Trovato, Vibha Patil, Maja Gehre, Kyung Min Noh

**Affiliations:** 1 Genome Biology Unit, European Molecular Biology Laboratory (EMBL), 69117 Heidelberg, Germany; matteo.trovato@embl.de (M.T.); vibha.patil@embl.de (V.P.); maja.gehre@embl.de (M.G.); 2 Collaboration for Joint PhD Degree between EMBL and Heidelberg University, Faculty of Biosciences, 69117 Heidelberg, Germany

**Keywords:** H3.3, H3, histone variants, histones, post-translational modifications, PTMs, chromatin, epigenetics

## Abstract

The systematic mutation of histone 3 (H3) genes in model organisms has proven to be a valuable tool to distinguish the functional role of histone residues. No system exists in mammalian cells to directly manipulate canonical histone H3 due to a large number of clustered and multi-loci histone genes. Over the years, oncogenic histone mutations in a subset of H3 have been identified in humans, and have advanced our understanding of the function of histone residues in health and disease. The oncogenic mutations are often found in one allele of the histone variant H3.3 genes, but they prompt severe changes in the epigenetic landscape of cells, and contribute to cancer development. Therefore, mutation approaches using H3.3 genes could be relevant to the determination of the functional role of histone residues in mammalian development without the replacement of canonical H3 genes. In this review, we describe the key findings from the H3 mutation studies in model organisms wherein the genetic replacement of canonical H3 is possible. We then turn our attention to H3.3 mutations in human cancers, and discuss H3.3 substitutions in the N-terminus, which were generated in order to explore the specific residue or associated post-translational modification.

## 1. Introduction

Histones are the building blocks of chromatin, participating primarily in DNA compaction within the nucleus of eukaryotic cells. Four main canonical histones (H2A, H2B, H3, and H4) constitute an octamer, which is surrounded by approximately 146 bp of DNA. This histone octamer-DNA complex composes a nucleosome, the basic unit of chromatin [1,2]. A common feature of the four main histone proteins is the presence of a structured core domain in close contact with the DNA and unstructured tails. Histone tails are essential parts of chromatin modifications whereby numerous enzymes contribute to various post-translational modifications (PTMs), such as acetylation, methylation, phosphorylation, and ubiquitination [3,4]. Individual modifications or specific combinations of PTMs are interpreted as a hallmark of defined chromatin environments. For instance, the tri-methylation (me3) of the 9th lysine residue (K9) on histone 3 (H3K9me3) is associated with gene silencing, and is enriched in heterochromatic regions [5]. H3K4me3 is generally enriched on the promoters and transcription start sites (TSS) of active genes [6,7,8]. H3K4me1 and H3K27ac are considered to indicate active enhancers [9,10,11,12]. All of these histone modifications are reversible, and in most cases, several enzymes are responsible for their deposition (i.e., writers) and removal (i.e., erasers). Furthermore, histone-modifying enzymes and the proteins that cooperate with them possess PTM recognition domains (i.e., readers), such as bromodomains and chromodomains, which recognize histone acetylation and methylation, respectively [13]. Histone PTMs are interpreted by chromatin-associated complexes, and can directly impact the surrounding epigenetic landscape by recruiting or inhibiting specific protein factors.

In addition to histone modifications, each canonical histone consists of several histone variants, with the exception of histone H4, where only one variant has been identified so far in humans [14,15]. Compared to the canonical counterparts, histone variants are diverse in both sequence and function, such that they can be localized at specific genomic regions. While canonical histones are encoded by multiple co-clustered genes expressed during the S-phase of the cell cycle, the variants are generally encoded by single-copy genes expressed throughout the cell cycle. In line with this, some variants are considered to be replacement histones because they can take the place of the canonical subtypes during development and differentiation, thereby becoming the predominant isoform in the differentiated cell [16,17]. The expansion of our understanding regarding the role of histone variants and uncovering their specialized functions is important for the characterization of chromatin dynamics.

## 2. Histone H3 Variants

The number of H3 variants is different among species, but two main subgroups are identified as replication-dependent and replication-independent variants. In human and mouse, H3.1 and H3.2 are the canonical subtypes. Their protein amounts peak in the S-phase and are deposited into chromatin coupled with DNA replication, and are thus referred to as replication-dependent histones. H3.1 and H3.2 are encoded by more than thirteen histone genes organized in clusters across different chromosomes [18,19,20]. These genes lack introns, their expression is coordinately regulated during the S-phase, and the resulting transcripts contain a characteristic 3′-stem-loop structure instead of a poly(A) tail [19,20,21]. 

Several histone H3 replacement variants exist in different species, with specialized functions. For example, the two testis-specific variants H3.4 (or H3.t) and H3.5 [22,23] have been reported as H3 variants exclusive to primates. Similarly, the expression of the H3.Y (or H3.Y1) and H3.X (or H3.Y2) replacement variants have been found in neuronal subpopulations in primates [24]. Despite unquestionable interest from both an evolutionary and molecular perspective, the current knowledge of these human and primate-specific H3 histone variants is still minimal, and is thus awaiting future research. Among the evolutionarily-conserved H3 variants, the function of the centromeric H3 variant CenH3 is intriguing. This variant is specifically localized at centromeres, and plays an essential role in regulating kinetochore assembly and chromosome segregation during mitosis [25,26]. Another H3 variant that is extremely conserved in eukaryotes, from yeasts to humans, is H3.3, which will be described in more detail in this review. 

## 3. Histone Variant H3.3

The histone variant H3.3 belongs to the replication-independent class of variants [27]. H3.3 is expressed across the cell cycle and predominates the H3 pool in post-replicative cells [28,29]. H3.3 is encoded by two conventional and independent genes (i.e., *H3f3a* and *H3f3b*) located on different chromosomes. Genetic studies in mice have shown that *H3f3a* and *H3f3b* double knockout is lethal due to severe developmental defects [30]. For single knockout mice, the results vary from normal and fertile mice to growth defects and reduced fertility [31,32]. The discrepancy between the studies [31,32,33,34,35] is likely due to differences in genetic background. Nevertheless, all of the studies conclude in a partial redundancy of H3.3 genes, and that one of the two H3.3 genes is required for mouse development, viability and fertility. The recent analysis aimed at clarifying the function and evolution of the two H3.3-coding genes in metazoans suggested that *H3f3b* resembles a more ancestral form of H3.3, while *H3f3a* could have arisen later in evolution, possibly due to duplication events [36]. The existence of two independent H3.3-coding genes seems to enable the fine-tuned expression of H3.3 genes in different cellular programs [36].

At the protein level, H3.3 differs only in five residues when compared to the canonical H3.1; four of them (i.e., A87, I89, G90 and S96) are placed in the histone fold domain, while the serine 31 is located in the N-terminal tail. The serine 31 is phosphorylated during mitosis, and this H3.3-specific PTM is enriched in the chromosomal regions that are adjacent to centromeres [37]. The four different residues in the core domain are critical for the recognition of H3.3 by dedicated protein chaperones: the histone cell cycle regulator (HIRA) and the death domain associated protein/alpha thalassemia/mental retardation syndrome X-linked (DAXX/ATRX) complexes. HIRA is responsible for the deposition of H3.3 in genomic regions characterized by an open chromatin state, such as the regulatory elements and gene bodies of actively transcribed genes [38,39,40,41]. DAXX/ATRX, on the other hand, are required for the deposition of H3.3 at telomeres, pericentromeric heterochromatin, and endogenous retroviral elements [38,39,42,43,44,45]. 

Covalent modifications of histones can occur in all H3 variants at the amino acid residues of the N-terminal tails and the internal histone fold domain. The question of whether H3.3 can show specific PTM patterns that are different from the canonical H3 variants, and if this helps in the definition of unique chromosomal domains, is still of great interest. Previous studies conducted in various laboratory models, ranging from *Drosophila* and mammals to *Arabidopsis*, showed that H3.3 is enriched in PTMs that correlate with an active chromatin state [46,47,48]. For instance, H3K4me, H3K9ac, and H3K36me1/2, which mark active transcription, are more enriched for H3.3 compared to H3.1 and H3.2. Some PTM enrichment on H3.3 showed co-occurrence, including K9ac-K14ac and K18ac-K23ac [48]. H3.3K9me3 was located at actively-transcribing repeats, for example, telomeres in stem cells [44].

## 4. Histone Residue Mutagenesis in Model Organisms: From Yeasts to *Drosophila*

Histone modifications are crucial elements in the regulation of gene expression, yet it is still unclear whether these marks have a direct impact on transcription or if their presence is a mere by-product of the transcriptional state of genes [49]. One approach to refine our understanding of histone PTMs and residues consists of the generation of histone mutants in model organisms. By mutating modifiable histone residues—such as lysine—into a non-modifiable one (alanine), a non-acetylated one (arginine), an acetyl-mimic (glutamine), or by mutating serine and tyrosine to a phosphor-mimic (glutamic/aspartic acid), we can test if the observed phenotypic effects are a direct consequence of the absence of specific residues, and of their chemical modifications. For example, K-to-R (lysine-to-arginine) substitution is effective in preventing modifications but retaining a positive charge on the exchanged histone residue. On the other hand, the relatively small and uncharged side-chain of alanine makes K-to-A substitutions ideal to completely abolish the target residue function. As a consequence, alanine substitutions are expected to have a more severe impact compared to arginine substitutions.

### 4.1. Yeast

Genome engineering with homologous recombination is highly effective in yeast, and it has been employed to generate systematic histone H3 and H4 substitution and deletion mutants in order to determine the contribution of each residue to nucleosome function. *Saccharomyces cerevisiae* carries only two gene copies for the entire pool of H3 [50]. Dai et al., 2008 for example, removed one H3 gene copy and exchanged the second gene. The systematic substation of all H3 residues with alanine was assessed in conjunction with all of the modifiable residues being replaced with amino acids mimicking an unmodified state [51,52]. Remarkably, only 47/407 point mutations—encompassing 40 residues—were essential for viability, and even long deletions of the N-terminal region of H3 were tolerable to a great extent. The primary amino acid substitutions which resulted in lethality were those that produced a net increase of negative surface charge [51]. Specifically, these were positively-charged residues on the DNA binding surface, as well as ‘semi-buried uncharged’ residues located in the nucleosome core. Of all the lethal point mutations, 35% in H3 and 38% in H4 showed a significant decrease in global protein abundance. From the pool of mutants that survived, mutants containing H3 tail deletions presented an inability to silence the transcription of ribosomal DNA. The observed phenotypic and lethality effects were interpreted to be a direct consequence of the absence of specific residues and their PTMs [51]. Another study with a similar approach showed, among other things, that substitutions at residues H3K56 and H3K115 in yeast resulted in hypersensitivity to drug-induced DNA damage, suggesting an essential role in the DNA damage response [53].

In order to address whether histone acetylation levels are altered during ageing, Western blot analyses on histone PTMs associated with transcription, DNA repair, and chromatin condensation were performed on *Saccharomyces cerevisiae* across its ageing. This revealed that H4K16 acetylation increased with age, whereas H3K56 acetylation decreased [54]. This age-dependent histone acetylation was further associated with transcriptional repression at specific loci near telomeres. The importance of H4K16ac in ageing was further consolidated by showing that the deletion of Sas2, a major H4K16 acetyltransferase, caused decreased H4K16ac at subtelomeric regions and increased yeast lifespan [54]. Similarly, in another study, a large-scale histone H3/H4 mutant library screening showed that H3K36 also plays a critical role in modulating yeast lifespan [55]. Again, the relationship was consolidated by showing that the loss of H3K36me3 through the deletion of the H3K36 methyltransferase Set2 reduced the lifespan, whereas increased H3K36me3 via the deletion of the H3K36 demethylase Rph1 increased the lifespan [55]. 

Collectively, studies such as these show that mutations on specific residues on H3/H4 tails in yeast can be essential for survival, and can affect transcription, chromatin organization, and the DNA damage response. In addition, the presence or absence of certain histone PTMs can also affect ageing. Hence, overall, histone PTMs collectively can have significant functional consequences in yeast [51,52,53,54,55,56].

### 4.2. Other Fungi

In fungal species, a number of gene pathways are involved in the formation of secondary metabolites such as penicillin, sterigmatocystin, and orsellinic acid. In one study, since viable strains of *Aspergillus nidulans* could be obtained from the systematic deletion of all H3 residues, this model organism proved highly useful in the investigation of the relationships between histone PTMs and secondary metabolite formation [57]. Through qRT-PCR and the genome sequencing of all of the mutant strains, this study showed that H3K14 acetylation is important for the production of secondary metabolites. The absence of this modification on gene promoters reduced the transcription of several genes involved in the biosynthesis pathways for penicillin, sterigmatocystin, and orsellinic acid. H3 acetylation appeared to function in two, non-mutually exclusive manners: (i) to provide a molecular tag for the recruitment of chromatin-modifying complexes, and (ii) to directly change the chromatin structure by weakening histone-DNA contacts. [57]. Hence, the presence or absence of histone PTMs can have larger downstream effects on transcriptional outputs. Another useful model organism is the *Neurospora crassa* fungus, because its genome contains a single H3 gene, *hH3*, on which nearly half of the approximately 40 amino acid residues are subject to PTMs [58]. One study altered ‘acetylatable’ residues to arginine or glutamine, and ‘methylatable’ residues to leucines in order to systematically study the effects of an absence of these modifications. Among others, a key finding was that the activity of a DNA methylation enzyme, DIM-5, was promoted by H3K9me3 and downregulated by H4K4me2 and H3S10ph. The modulation of the activity of DIM-5 had downstream transcriptional consequences. Hence, specific histone PTMs can affect transcription via the modulation of specific enzymes [58].

### 4.3. Fly

The presence of multiple gene copies coding for the same histone protein poses a severe obstacle to mutational studies of histones in metazoans. Among all multicellular organisms, this goal has been accomplished so far only in *Drosophila melanogaster*. In this regard, the fruit fly represents an exception among metazoans, because all canonical histone genes are organized in tandem repeats located at a single chromosomal locus [59]. Various genetic tools, such as plasmid and bacterial artificial chromosome (BAC)-based transgenes, and more recently clustered regularly interspaced short palindromic repeats (CRISPR)-Cas9, have been used to replace the wild-type genes with mutated versions [59,60,61,62,63]. This landmark study reported that a lysine-to-arginine substitution at the H3K27 residue (H3K27R) could phenocopy homeotic defects observed in polycomb repressive complex 2 (PRC2) mutants, suggesting a direct role of PRC2-mediated H3K27me3 in this process [63]). A similar substitution at the H3K36 residue (H3K36R) revealed an essential role of H3K36 for *Drosophila* viability [59], and a follow up study on H3K36R substitution in *Drosophila* cells found global dysregulation of mRNA, suggesting a critical post-transcriptional role for H3K36me3 [64]. Furthermore, a recent systemic study testing alanine substitution of many modifiable histone residues expanded the pool of histone mutants related to *Drosophila* viability (e.g., H3K4; H3K9; H3K14; H3K27; H3K36). Among the mutants that survived in the early developmental stages, many show reduced fertility (e.g., H3T3A; H3K56A), susceptibility to DNA damage (e.g., H3K56A), and defects in gene silencing (e.g., H3K9A; H3K23A) [61]. Therefore, it is likely that, with the increasing complexity of multicellular organisms, histone residues have acquired new roles in DNA-related processes. This is especially relevant when considering the importance of cell-specific and developmental stage-specific gene expression programs which need tight regulation during the life cycle of complex, multicellular eukaryotes [65]. It is also worth pointing out that these studies mutated the canonical histones in the presence of wild-type H3.3, which could compensate if the targeting modification is enriched in the H3.3 variant.

Overall, these studies have shown that histone functionality has gained complexity during the evolution of multicellular organisms, and many modifiable residues on histone tails are essential for viability and transcriptional regulation in *Drosophila melanogaster* compared to *Saccharomyces cerevisiae.*

## 5. Histone PTM Studies through the Perturbation of Chromatin Factors

Due to the multi-copy nature of the canonical histone gene clusters spread throughout different chromosomes, histone mutagenesis studies in mammals have been considered almost impossible. Traditionally, the genes encoding histone-modifying enzymes were removed in order to assess the phenotypic consequences of the absence of the catalysed chemical modifications. This knockout approach has been illuminating. For instance, the deletion of the catalytic subunit of the PRC2 known as EZH2, and that of other PRC2 subunits, has been crucial to the characterization of the repressive role of the H3K27me3 mark, both in model organisms and cell cultures [66,67,68,69,70,71,72]. However, histone writers are often part of larger chromatin complexes, and the deletion of one component can lead to the disruption of such complexes. Therefore, the loss of functions might be independent from the catalytic activity of the deleted writers [73,74]. This issue can be overcome by introducing mutations that abolish only the catalytic activity of the histone writers, without affecting their complex formation, and this approach has already been proven to be valuable in several studies [72,73,74,75,76,77]. In addition, growing interest in ‘epigenetic drugs’ (e.g., EZH2 inhibitors, bromodomain antagonists, histone deacetylase inhibitors) and the possibility to modulate the activity of epigenetic factors for therapeutic purposes, have advanced the use of small-molecules as an alternative and complementary approach to the study of histone-modifying enzymes and chromatin readers [78,79].

The genetic deletion and chemical perturbation of histone modifiers aimed to infer the role of histone PTMs can be effective when a specific histone mark is known to be deposited by only one enzyme. However, a one-to-one relation between writers and histone marks rarely takes place, and most PTMs can be deposited by several distinct enzymes (e.g., H3K9 acetylation by p300/CBP and GCN5/PCAF; H3K9 methylation by G9a, EHMT1, SUV39H1/2 and others [3,5]). Even if the levels of a specific PTM are severely reduced—genome-wide—upon the deletion of dedicated enzymes, compensatory mechanisms may help to retain the modification on specific genomic locations via non-canonical or as-yet unidentified pathways [80]. 

**Figure 1:**
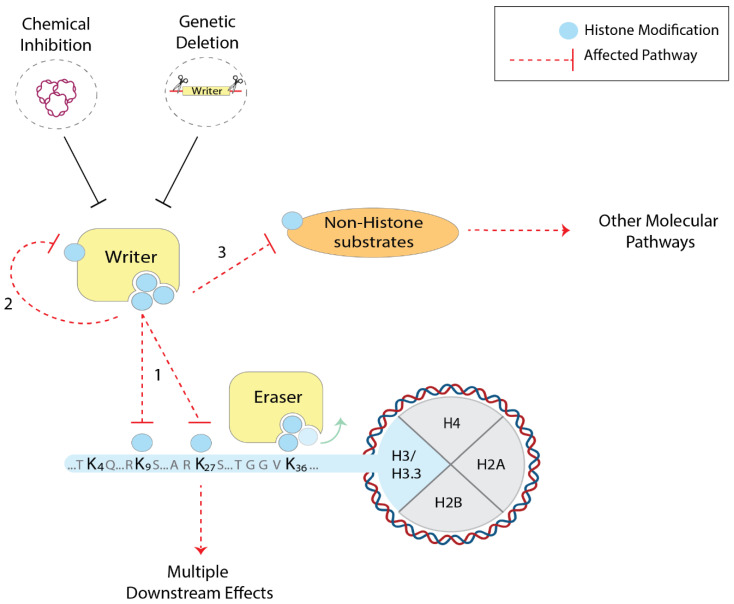
Chemical and genetic perturbation of chromatin factors to investigate histone residues and PTMs in mammals. Histone writers (yellow) can often post-translationally modify multiple histone residues (1), but also themselves (2) and other non-histone substrates (3). As a consequence, when employing the chemical and genetic perturbations of such enzymes, multiple cellular pathways can be affected (red dashed arrows), making it difficult to infer the role of histone PTMs.

Another concern is the high functional promiscuity of most histone writers. Many enzymes that catalyse the deposition of chemical groups can not only modify multiple histone residues (e.g., the acetylation of H3K27, K18, 14 and others by p300/CBP; the methylation of H3K9 and K56 by G9a [3,4,5]), but they can often modify themselves and other non-histone substrates. For example, histone acetyltransferases (HATs) like p300/CBP, as well as histone methyltransferases (HMTs), can post-translationally modify transcription factors and many other nuclear proteins [81,82,83,84]. Hence, the results that aim to establish a direct link between the genetic deletion of histone modifiers and the role of specific histone PTMs must be interpreted with caution. When chemical perturbations are employed as an alternative approach, substrate specificity is an additional problem. Poor target selectivity both in vitro and in vivo has been reported previously, which may interfere with other cellular pathways [85]. It can, therefore, be challenging to infer the role of histone modifications through the perturbation of histone writers (Figure 1).

## 6. H3.3 Mutations in Cancer

A breakthrough in the study of histone mutations occurred when several groups reported an alteration of histone H3 genes, including H3.3, as a cause for specific cancers [86,87,88,89,90,91,92,93,94].

Distinct missense mutations appeared in H3.3-coding genes with different frequencies in either *H3f3a* or *H3f3b*. For instance, heterozygous K27M (lysine-to-methionine) or G34R/V (glycine-to-arginine/valine) mutations, preferentially occurring on *H3f3a*, were found in pediatric high-grade gliomas, whereas K36M mutation in chondroblastoma was predominant on *H3f3b* [87,90]. K27M glioma mutation was also observed in one of the canonical H3 genes, which showed less metastatic recurrences than those found in H3.3 genes [90,92,93]. These results imply that a small amount of mutation within the total histone H3 pool was enough to promote the cancers via the gain of function. 

By combining in vitro and in vivo approaches, the oncogenic mechanism of the K27M and K36M mutations has been elucidated and extensively reviewed [95,96]. Briefly, the lysine-to-methionine substitutions reported in diffuse intrinsic pontine gliomas (i.e., K27M) and chondroblastomas (i.e., K36M) share a similar molecular mechanism. Both mutations induce a global decrease in the methylation levels of the corresponding lysine residue on wild-type histone H3. Biochemical and structural studies revealed that K27M has a high affinity for the catalytic subunit EZH2 of the polycomb repressive complex 2 (PRC2). K27M has been proposed to compete with lysine 27 on histone H3, sequestering PRC2 and thus preventing the propagation of the repressive H3K27me3 mark [97,98,99].

Over the years, the PRC2 sequestration model has been complicated by conflicting observations that, despite the high-affinity binding between purified PRC2 protein and H3K27M, they often do not co-occupy chromatin, and some H3K27me3 remained at endogenous loci [96,100,101,102]. Recent studies have clarified that H3K27M inhibits PRC2 in the presence of the H3K27me3 mark, a condition in which PRC2 is allosterically-activated by its own substrate [98,103]. H3K27M preferentially interacts with and stalls PRC2 at CpG islands, where H3K27me3 is retained [104]. Different levels of PRC2 inhibition and H3K27me3 loss are also coupled with the source of the mutant. H3.3K27M is generally enriched in active chromatin, and H3.1K27M diffuses across the genome following H3.3 and H3.1 distribution, respectively. This variation appears to be associated with the different subtypes of pediatric glioma occurring by H3.3K27M and H3.1K27M [105,106]. Several studies have also pointed out that K27M mutation could affect H3K27ac levels, resulting in aberrant gene expression and enhancer dysfunction [100,107]. This implies that K27M mutation can disrupt both the methylation and acetylation states of the corresponding lysine residue; therefore, careful dissection is necessary when the outcome of the histone mutant is interpreted. 

The function of K27M and G34R/W H3.3 mutations have been further investigated in disease-relevant systems using knockdown approaches (K27M knockdown in a xenografted tumor; G34W knockdown in primary tumor tissue) and CRISPR-mediated knock-in (isogenic reverting K27M and G34R to K27 and G34, respectively) [108,109,110,111]. Disease-relevant systems also include cancer mouse models expressing H3.3K27M in neuronal stem cells [102,112] and in vitro derived human neuronal progenitor cells (NPCs), in which a K27M or wild-type H3.3 was exogenously expressed together with other oncogenes observed in H3.3K27M mutant cancer cells [113]. The advance of single-cell genomics has also applied and identified the gene expression programs of cells derived from patient biopsies [114,115]. 

The oncogenic mechanism of the K36M mutation found in chondroblastomas was similar to K27M, wherein it inhibited histone H3K36-specific methyltransferases (e.g., NSD1/2; SETD2), reducing the global level of H3K36 methylation [116,117,118]. Notably, the H3K36me2/me3 reduction was accompanied by an increase of H3K27me3 in the intergenic regions [116], thus supporting the previous observations that H3K36 methylation restrains PRC2-mediated H3K27me3 and prevents its spread across genomic regions [119,120].

Unlike lysines 27 and 36, glycine 34 on histone H3 is not post-translationally modifiable, implying the functional role of the histone residue itself. K27/36M mutation evenly occurred on canonical histone H3 genes, but glycine 34 missense mutations exclusively appeared on H3.3-coding genes, both in gliomas and bone tumors. The ectopic expression of H3.3, harbouring either glycine34-to-leucine or glycine34-to-tryptophan substitution, resulted in reduced K27 and K36 methylation levels [121]. Similarly, glycine34-to-aspartate/valine/arginine (H3.3G34D/V/R) mutations occurring in subtypes of gliomas reduced the H3K36 methylation [122]. Thus, glycine 34 mutations affect the modification status of nearby lysine residues. As previously suggested, the reason is likely that the presence of amino acids with bulky side chains at position 34 may generate a steric hindrance, thus keeping the SET domain containing 2 (SETD2) activity from the H3 N-terminal tail and reducing the H3K36 methylation. In addition, H3.3G34 mutations disrupted the recruitment of the protein factors involved in DNA mismatch repair pathways, increasing the susceptibility to additional mutations observed in cancer cells [122]. 

Notably, a recent study revealed that H3.3G34 mutations particularly affect SETD2 activity, which in turn results in reduced H3K36me3, with a concomitant increase of H3K27me3 in-cis [123]. More specifically, in mouse mesenchymal stem cells expressing a H3.3G34W or G34R mutant, the H3K36me3 loss/H3K27me3 gain occurred at a subset of enhancers, and was associated with reduced enhancer activity and downregulated proximal genes. By employing the chemical and genetic perturbation of SETD2 and PRC2, the authors validated the target gene expression changes observed in the H3.3G34W mutant [123].

Voon et al., 2018 complemented these results by showing that the H3.3G34R mutation increased the H3K36me3 signal at specific genomic loci by inhibiting histone demethylase enzymes [124]. When a heterozygous *H3f3a* G34R mutation was introduced in mouse embryonic stem cells (mESCs), H3K36me3 signals were increased in the genomic regions bound to lysine-specific demethylase 4 (KDM4)-A/B/C. The H3K9me3 signal was also affected in a similar way, which is consistent with the fact that these demethylases act on both lysines. The authors suggested that KDM4 enzymes preferentially bind G34R mutant H3.3, and that the presence of an arginine in place of the glycine inhibits their catalytic activity [124]. 

Overall, these studies aimed to understand the molecular mechanisms of oncogenic mutations, illustrating that histone mutations intricately affect chromatin pathways by altering the activity of histone-modifying enzymes (Table 1). This molecular perspective will be expanded as more cancer-associated histone mutations are identified at a lower frequency on other H3.3 residues, including in the globular domain [125]. Histone H3.3 mutagenesis will complement studies focused on chromatin writers, readers, and erasers by answering how histone residues and their PTMs participate in the complex epigenetic mechanisms that regulate gene expression.

## 7. Histone H3.3 Mutagenesis: A Way to Study Histone Residues and PTMs in Mammals

**Figure 2:**
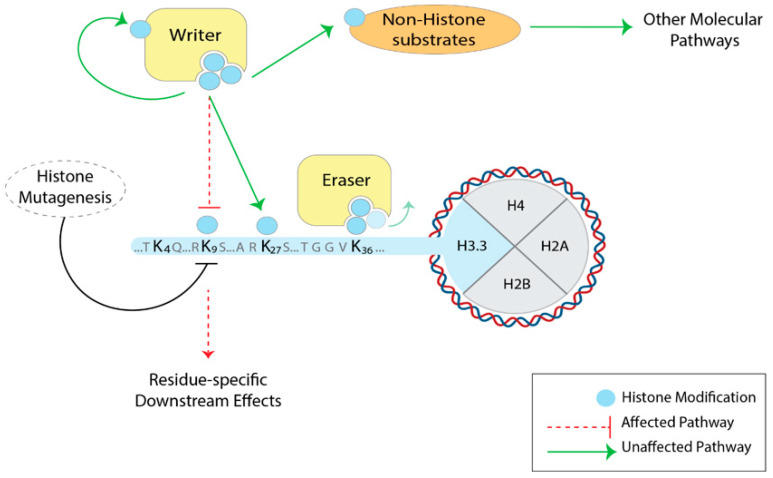
Histone H3.3 mutagenesis to study histone residues and PTMs in mammals. By mutating specific residues of H3.3, the downstream effects are expected to be more residue-specific. The activity of the histone-modifying enzymes (yellow) themselves is not affected: the deposition of the modifications on other histone residues, as well as other chromatin pathways, is preserved (green arrows). Note that H3.3 mutagenesis—such as lysine-to-alanine—would disrupt all PTMs (acetylation, methylation, ubiquitination), so it does not address PTM-specific effects.

As we learned from histone mutational studies carried out in unicellular and multicellular eukaryotes, deletions of the entire N-terminal tail or the mutation of individual residues do not affect yeast viability [51], but showed a dramatic effect on *Drosophila* development, often inducing lethality across the developmental stages of the fly [59,64]. Therefore, it is crucial to investigate the function of histone tail residues in complex eukaryotes. However, due to the multi-copy and multi-chromosome distribution of the canonical histone genes, it is extremely challenging to implement a tool for canonical histone exchange in mammals. The genes of replication-independent histone variants, such as H3.3, exist in two copies outside the histone clusters [38], and recent advances in genome engineering techniques (i.e., CRISPR-Cas9) have made targeted mutations inside these genes feasible, opening up the possibility for systematic histone mutations in the mammalian system (Figure 2).

Histone H3.3 mutagenesis has become a powerful tool to probe the function of histone residues in mammalian cells, wherein three distinct approaches have been implemented so far: (i) the exogenous overexpression of H3.3 mutants, (ii) the generation of H3.3 knockout cell lines and the expression of exogenous histone H3.3 mutants, and (iii) the editing and mutation of endogenous H3.3-coding genes. All three approaches have proven valuable, as detailed in the next section. We will focus on the substitutions in the N-terminus of H3.3 residues which are associated with crucial post-translational modifications, and do not cover the mutations in globular regions related to H3.3 deposition pathways by specific histone chaperones.

## 8. H3.3 Mutagenesis beyond Cancer

### 8.1. K9M and K36M

The pioneering work from Lewis et al., 2013 showed that K-to-M substitutions, which inhibit SET-domain-containing histone methyltransferases (HMTs), can serve as a tool to study histone lysine methylation. K9M substitution, in addition to K27M and K36M mutations [97,116], is capable of inhibiting the activity of K9-specific HMTs such as G9a and SUV39H1 [97]. This mechanism is conserved throughout evolution, as H3.3 K-to-M mutants have been exploited in *Drosophila* in order to better understand histone methylation pathways [62]. 

More recently, inducible K-to-M mutations were applied in mice to investigate histone methylation pathways across development and differentiation [126]. Doxycycline-inducible H3.3 K9M and K36M mutations showed a defective differentiation of mESCs to embryoid bodies (EBs). Knockdown experiments of H3K36-specific HMTs (i.e., Nsd1 or Setd2) partially recapitulated the phenotype for the K36M mutation, whereas the knockdown of H3K9-specific HMTs (i.e., G9a or Setdb1) did not result in differentiation defects similar to the ones observed for the K9M mutant [126]. This result emphasizes that histone mutagenesis can help us to study the role of histone PTMs when the perturbation of histone-modifying enzymes is ineffective for several reasons (e.g., the redundancy of K9-specific HMTs). Transgenic mice were then derived from the mESC lines carrying either one or the other mutation. Upon the induction of the H3.3 mutant transgene expression (at 6–8 weeks of age), the K9M and K36M mutant mice displayed a residue-specific array of developmental defects. Focusing on hematopoiesis, the K9M and K36M mutations resulted in erroneous cell fate decisions during the hematopoietic stem and progenitor cell differentiation [126]. Therefore, K-to-M mutations successfully assessed the impact of histone methylation changes on physiological processes. Such an experimental approach opens a new way to probe the contribution of histone residues and PTMs in complex biological processes. Lysine-to-methionine substitutions are helpful specifically when focusing on histone methylation, but the dominant gain-of-function effect of such mutations on histone methyltransferases might mask other phenotypic outcomes that are independent of the methylation state of the residues. For this reason, other amino acid substitutions have been recently exploited in order to address the function of histone residues in mammals.

### 8.2. K4A/R and K36A

Our group investigated the role of H3.3K4 and K36 residues by introducing K-to-A mutations that completely disabled the function of the target residue [127]. Using CRISPR-Cas9 in mESCs, we deleted the *H3f3a* gene and introduced alanine substitution on the endogenous *H3f3b* gene; thus, the entire H3.3 pool contained mutation [128]. The two mutations did not affect the self-renewal in mESCs, but—upon the induction of the in vitro neuronal differentiation—the K4A mutant showed significantly reduced cell numbers at the EBs and NPCs stages, and failed to differentiate into glutamatergic neurons. The K36A mutant differentiated into neurons, despite reaching a higher cell density compared to the control. These differences were reflected in the transcriptional changes. The K36A altered the gene expression profiles mainly in neurons, which is consistent with an increased contribution of H3.3 to the total H3 pool in post-mitotic neurons [28]. The K4A mutant caused widespread gene expression changes in both mESCs and neurons. This result proves that single amino acid substitutions on H3.3 can be effective, even if this variant accounts for a small portion of the total H3 pool in fast-dividing cells such as mESCs [28]. 

**Figure 3:**
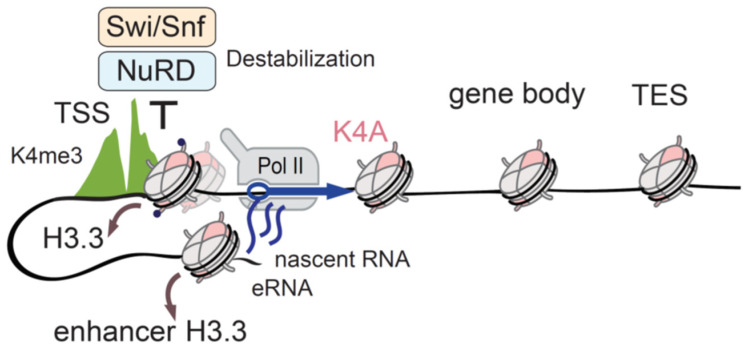
Identifying H3.3K4 function with endogenous H3.3 mutation. The unstable H3.3K4A (pink) mutant is depleted from TSSs and enhancers (brown arrows), but H3K4me3 levels (green) are unchanged due to the existence of the canonical histone H3.1/H3.2. The recruitment of the chromatin remodelers NuRD (light blue) and Swi/Snf (light orange) to the regulatory regions is perturbed in H3.3K4A ESCs, because these complexes require the K4 residue for binding to the H3 histone tail. The disturbed remodeler occupancy results in the misregulation of Pol II activity (blue arrow), and aberrant transcription from TSS and linked enhancers. H3.3 mutation helped with the clarification of the functional role of the H3K4 residue in chromatin regulation and transcription. Transcription start sites (TSS); Transcription end sites (TES); Enhancer RNA(eRNA).

The profound effects on gene expression that arise in a single histone residue substitution imply extensive changes in the chromatin environment. Indeed, the K4A mutant was depleted at promoters and enhancers, but maintained other genomic regions in mESCs, suggesting that the H3.3K4A is less stable than wild-type H3.3 at the regulatory regions. Chromatin remodelers in part mediate this, as the K4A mutation disrupted the binding of the components of the nucleosome remodelling deacetylase (NuRD) and switch/sucrose non-fermentable (SWI/SNF) chromatin remodelling complexes. In the presence of the H3.3K4A mutant, the occupancy of the core components of these remodelling complexes was reduced at the TSS and active enhancers, which in turn increased the nascent RNA levels and RNA-polymerase II (RNA-Pol II) occupancy (Figure 3).

The H3.3K36A mutant mESCs showed only minor gene expression changes, and neither the H3.3 deposition nor the RNA-Pol II activity was affected. However, the H3K36me2 levels were decreased in the intragenic regions, causing a concomitant spread of the H3K27me3 mark [127], partially recapitulating the observations from the H3.3K36M mutational studies [116].

Chromatin remodelling complexes are essential regulators of DNA-templated processes, and they often rely on the presence of histone PTMs to recognize their nucleosome substrates [129]. This recognition is mediated by protein domains that are specific for the defined PTMs [13]. Through the application of H3.3 mutagenesis, we found that unmodified K4 residue itself is crucial for the recognition and binding of NuRD and SWI/SNF complexes to nucleosomes in the mammalian genome. This finding is consistent with the results reported by Kraushaar et al., 2018. They engineered mouse embryonic fibroblasts to express a HA-Flag-tagged H3.3, either wild-type or with lysine-to-arginine substitutions at K4, K9, K27, or K36. By means of immunoprecipitation coupled with mass-spectrometry, they found that K4R (and to a lesser extent K9R) mutation reduced the number of protein partners, especially the components of the NuRD complex relative to the wild-type control. Consistently, H3.3 knockdown decreased the recruitment of NuRD subunits, both genome-wide and at the TSSs of the actively-transcribed genes [130].

### 8.3. K27R

So far, attempts to investigate the functionalities of histone PTMs in enhancers have been focused on mutations or knockout/knockdown studies of histone writers/readers [76], or mutations of chromatin reader domains [131]. The histone variant H3.3 is enriched at the active enhancer and promoter elements, indicating a rapid histone turnover [132,133,134]. Since H3.3 is also enriched for active histone PTMs such as H3K27ac [46,48], Zhang et al., 2020 recently applied H3.3 mutagenesis in order to probe the contribution of H3K27ac to the enhancer function [135]. Using CRISPR-Cas9, they mutated all endogenous H3.3-coding genes to K27R. The H3K27ac signal was severely depleted at the active enhancers in the mutant mESCs compared to the wild-type, and a minor reduction was also detected at the promoters. Notably, despite the severe loss of H3K27ac at the enhancers, the gene expression was only modestly affected in this experimental setup. Among the differentially-expressed genes in the K27R mutant compared to the wild-type, ~70% were downregulated. The analysis of enhancer-promoter connections did not reveal any correlation between H3K27ac loss at the enhancers and the misregulation of the gene expression, since the enhancers of both up- and down-regulated genes displayed decreased H3K27ac. Furthermore, the chromatin accessibility at the enhancers and the RNA-Pol II occupancy at the TSSs were unaltered [135].

These results in mESCs are concordant with another recent study conducted in *Drosophila*, in which H3.3K27R mutants showed a reduction in H3K27ac levels, but only a few transcriptional changes [136]. Related to the H3K27ac changes and transcriptional outcome, a previous study of CBP/p300 catalytic inhibition led to the global loss of H3K27ac, but induced a few transcription changes [137]. Similarly, MLL3/4 catalytic mutations induced the global reduction of H3K27ac, with minor effects on gene expression [73,74].

Overall, these studies indicate that H3K27ac is dispensable for enhancer activity and transcription regulation. Given that H3K27ac is widely considered to be a mark of active enhancers, these results demand a reconsideration of the role of H3K27ac in gene expression regulation. One possibility is that H3K27ac levels might affect gene expression upon the challenging condition to the cells, rather than the steady state. The other possibility is that the acetylation at other lysines located in the H3 tail (e.g., H3K14, K18, K23) and the H3 globular domain reported to occur at enhancer elements [138,139,140] could compensate the H3K27ac. We can hence speculate that there could be redundancy among acetyl-lysines at enhancers, and H3K27ac might play a less crucial role in regulating gene expression.

### 8.4. S31A/E

In another recent study, Martire et al., 2019 introduced mutations to the H3.3S31 residue unique to the H3.3 variant, and investigated whether the H3.3 deposition at the enhancer elements represents a fundamental prerequisite for enhancer function [141]. They found that the phosphorylation occurring on serine 31 can boost the p300 activity at the enhancer elements. In H3.3 knockout mESCs, the H3K27ac mark was globally reduced, and the histone acetylation at K27 (as well as K18, K64 and K122) was particularly affected at distal enhancers. However, neither the chromatin accessibility nor p300 recruitment at these genomic regions were affected, and the loss of H3K27ac in the H3.3 KO cells did not result in global reductions in gene expression. The exogenous expression of H3.3 mutants in H3.3 KO mESCs that were able to mimic phosphorylation (i.e., serine-to-glutamic acid: S31E) or unable to be phosphorylated (i.e., serine-to-alanine: S31A) at position 31 revealed that only the S31E mutant was capable of restoring the H3K27ac levels [141]. In EBs differentiated from H3.3 KO mESCs, a reduction of the H3K27ac signal was observed at the specific enhancer elements which usually acquire this mark during the differentiation of wild-type mESCs. Moreover, the H3K27ac loss at these differentiation-specific enhancers was associated with the decreased expression of the nearest genes [141]. These results suggest that H3K27ac loss might not affect enhancer activity and steady-state transcription, but can impact stem cell differentiation, perhaps driving a defective activation of the regulatory elements that are necessary during the process.

The central role of H3.3 serine 31 in gene expression regulation and development has been investigated in *Xenopus laevis*, as well [142]. By systematically mutating H3.3 residues, the authors found that H3.3 serine 31 is essential for *Xenopus* gastrulation, and—using phosphomimetic mutants—they also linked serine 31 phosphorylation to H3K27ac [142]. In another study, Armache et al., 2020 investigated the role of H3.3S31ph in lipopolysaccharide-induced transcription in macrophages [143]. They found that H3.3 serine 31 phosphomimetic mutants and H3.3S31ph recruit SETD2 in order to drive rapid stimulus-induced gene expression [143]. 

Lastly, histone H3.3 mutagenesis can also be used to help define the function of newly discovered histone PTMs. Two recent studies, in particular, described the serotonylation and dopaminylation of glutamine 5 on the H3 histone N-terminal tail as novel histone marks playing a role in transcription factor binding and neuronal transcriptional plasticity, respectively [144,145]. In both studies, the expression of H3.3Q5A mutant proteins that prevent the deposition of such modifications was crucial in order to characterize the downstream effects of these novel histone PTMs on transcriptional regulation [144,145]. Taken all together, histone H3.3 mutagenesis represents a powerful tool to investigate the role of histone residues in mammals (Table 2). By assessing the downstream effects of H3.3 mutants’ expression on complex biological processes, we can expand our understanding of the ways in which histone residues and their PTMs contribute to gene expression regulation.

## 9. Conclusions and Future Perspectives

The histone variant H3.3 accounts for a relatively small portion of the total H3 pool in dividing cells (e.g., ~20% of the total H3 in mESCs [28]). However, single amino acid substitutions of H3.3 exhibit major impacts on gene expression, the cell cycle, cell differentiation, cellular homeostasis, and organism development [126,127,141,142,143,146]. This could be, in part, due to the tightly regulated deposition of H3.3 at specific genomic regions such as promoters, enhancers, and several repeat elements [39]. Therefore, studies that aim to characterize histone PTM function using H3.3 mutations should consider that the deposition pattern of H3.3 itself could favor the mis-regulation of specific chromatin pathways over others. Similarly, mutations of specific residues that are preferentially post-translationally modified on H3.3 could cause more dramatic effects on the epigenetic landscape and transcriptional programs.

In H3.3 mutagenesis, the chemical properties of the amino acids being substituted may generate profound differences in the outcome. As described above, some histone residue mutations, like lysine-to-methionine substitutions, can show a dominant phenotype due to the inhibitory effect on HMT enzymes, and potentially other biological processes. Mutations to amino acids that mimic persistent PTMs on histone residues represent another type of gain-of-function substitution that can be extremely helpful when focusing on specific histone modifications. The use of modification-mimetic mutations of histone residues has been applied to organisms such as yeast and *Drosophila* [147,148], and the recent results reported using phosphomimetic serine 31 H3.3 mutants represent a good example of the way in which the same approach can be applied to the study of histone PTMs in mammalian cells [141,143]. Apart from the phosphomimetic serine-to-glutamic acid substitution, lysine-to-glutamine substitutions have also been used to mimic acetylation [149,150,151]. A more complex way to introduce persistent post-translational modifications could rely on the ‘expansion of the genetic code’. This consists of the generation of cell lines that are capable of producing proteins with synthetic unnatural amino acids [152,153]. Using this strategy, Elsässer et al., 2016 presented an innovative system to study histone PTMs wherein they generated mammalian cell lines genetically encoding acetyl-lysines at specific residues of the H3.3 histone variant, and uncovered functions of several H3.3 acetylated lysines in regulating transcription, including K37 and K56 [154]. Therefore, the combination of H3.3 mutagenesis with approaches that allow the introduction of synthetic chemical modifications [155] can provide powerful means for the study of histone PTMs in mammalian cells, and may open experimental avenues that have not yet been explored.

Loss-of-function mutations of histone residues, like lysine-to-arginine or -alanine substitutions, prevent the deposition of certain PTMs on the targeted residues. We extensively discussed that these mutations are hence useful in order to understand the ways in which the loss of histone modifications can affect biological processes such as gene expression, without the need to act on histone-modifying enzymes. Beyond the widespread impact of H3.3 mutations on gene expression, there are also consequences for other nuclear processes, such as DNA damage repair [122,156]. Despite the indication of the data collected so far that H3.3 mutagenesis is effective in determining genomic region-specific reductions of histone PTMs, it is important to take into account the fact that modifications on canonical H3 proteins might compensate for the loss of H3.3 residues. While further studies will be necessary in order to investigate the extent of such mechanisms, we speculate that, even if it were possible, a complete loss of a certain H3 histone residue and its associated modifications may cause phenotypes too severe to allow the characterization of the contribution of histone residues in mammals. Studies conducted in *Drosophila* showed that loss-of-function mutations of residues of the H3 N-terminal tail, such as K4, K9, K27 and K36, among others, are lethal at different developmental stages [61]. Therefore, the retained modifications on the canonical H3 histones do not necessarily represent a disadvantage. On the contrary, mutating only H3.3 might offer an experimental advantage by ‘killing’ histone modifications in a variant-selective manner.

With the advancing research in histone PTMs, histone mutations and engineered histone residues must be considered as fundamental tools to shed light on the contribution of histone residues in organismal biology and pathology. We anticipate that future research exploiting H3.3 mutations will allow us to uncover new roles of histone residues and their modifications.

## Figures and Tables

**Table 1:** Molecular mechanisms of histone H3 mutations in cancers.

Mutation	Genes	Type of Cancers	Molecular Outcomes/Mechanisms	References
K27M	H3F3A; HIST1H3B	Pediatric Glioblastomas	Inhibition of PRC2; reduction of H3K27me	[86,87,88,89,91]
K36M	H3F3B; HIST1H3C	Chondroblastomas	Inhibition of NSD1/2, SETD2	[90,92,93,116,118]
G34R/V	H3F3A	Pediatric Glioblastomas	Affect SETD2 activity (possibly due to steric hindrance); decrease of H3K36me3 and increase of H3K27me3; inhibition of demthylases	[87,90,91,94,122,123,124]
G34W/L	H3F3A	Giant Cell Tumors of the Bone		
G34W/R	H3F3A	Osteosarcomas		

**Table 2:** Histone H3.3 mutants in mammalian systems.

Mutation	Model Systems	Phenotypic Effects	Molecular Outcomes/Mechanisms	References
K4A/R	mESCs; in vitro derived neurons; MEFs	Impaired in vitro differentiation	Extensive gene expression changes; affect binding of NuRD and SWI/SNF remodeler complexes; H3.3 depletion at TSSs and enhancers; dysregulation of Pol II activity	[127,130]
K9M	mESCs; HSPCs; *Mus musculus*	Impaired in vitro differentiation; impaired hematopoiesis in vivo	Increased chromatin accessibility; global reduction of H3K9me; inhibition of H3K9-specific HMTs (i.e., G9a or Setdb1)	[97,126]
K27R	mESCs	N/A *	Extensive reduction of H3K27ac at enhancers and modest reduction at TSSs	[135]
S31A/E	mESCs	N/A *	H3.3S31ph boosts p300 activity at enhancers	[141]
K36A	mESCs; in vitro derived neurons	Higher cell density of neuronal networks	Gene expression changes in neurons; global reduction of H3K36me2/3; increase and spread of H3K27me3	[116,126,127]
K36M	mESCs; HSPCs; *Mus musculus*	impaired in vitro differentiation; poor body condition and early lethality; impaired spermatogenesis, intestine cells differentiation and hematopiesis in vivo	Increased chromatin accessibility; global reduction of H3K36me3; increase and spread of H3K27me3	

N/A (not available). * The authors did not report any major phenotypic effect as a consequence of the introduced mutation (e.g., morphology, differentiation, etc.).
